# Supplementary material for: Multiplex Protein Biomarker Profiling in Patients with Familial Hypercholesterolemia
Source: Genes (Basel). 2021 Oct 12;12(10):1599. doi: 10.3390/genes12101599 (PMC8535274; doi:10.3390/genes12101599)
Supplement: Supplementary file 1 [file genes-12-01599-s001.zip › genes-1387826-supplementary.pdf]

Table S1 Plasma levels of proteins differentiating before and after therapy.

**a/ LLD$^+$/AF$^-$**				
**Protein biomarker**	**Mean Linear ddCq (95%CI)**		**FC**	***P corr***
	**Before**	**After**		
TM	429 (402-457)	387 (365-408)	-1.109	1.6 x 10^{-3}
DKK-1	950 (742-1159)	758 (589-926)	-1.267	1.7 x 10^{-3}
CCL3	19 (15.2-22.9)	15 (12.8-17.3)	-1.234	1.8 x 10^{-3}
ST2	8.03 (6.2-9.9)	10.1 (7.2-12.9)	1.219	3.7 x 10^{-3}
CD4	7.4 (6.8-8.2)	6.7 (5.9-7.4)	-1.116	5.1 x 10^{-3}
PDFG subunit B	1439 (1166-1711)	1148 (807-1486)	-1.420	5.6 x 10^{-3}
AGRP	21.1 (18.7-23.6)	17.6 (15.4-19.8)	-1.206	6.6 x 10^{-3}
IL18	299 (246-352)	255 (213-297)	-1.152	6.8 x 10^{-3}
THPO	8.6 (7.7-9.4)	7.4 (6.7-8.2)	-1.154	8.3 x 10^{-3}
LOX1	103 (87-119)	88 (75-101)	-1.157	9.5 x 10^{-3}
**b/ LLD$^+$/AF$^+$**				
**Protein biomarker**	**Mean Linear ddCq (95%CI)**		**FC**	***P corr***
	**Before**	**After**		
LDLR	25.7 (16.5-34.9)	12.1 (8.2-16.1)	-2.222	1.6 x 10^{-6}
OPN	191 (137-245)	367 (284-451)	1.975	4.3 x 10^{-6}
PCSK9	14.9 (10.1-19.6)	10.6 (6.1-15.1)	-1.544	1.1 x 10^{-4}
MMP-3	111 (79-143)	77 (60-94)	-1.372	1.9 x 10^{-4}
COL1A1	17.7 (14.4-21)	22.8 (18.8-26.7)	1.289	2.3 x 10^{-4}
GDF-2	14.5 (11.6-17.4)	8.7 (6.4-11.1)	-1.738	3.7 x 10^{-4}
KLK6	17.1 (14.8-19.4)	23.2 (19.9-26.6)	1.356	4.1 x 10^{-4}
CTRC	1417 (1201-1633)	967 (751-1183)	-1.510	4.3 x 10^{-4}
IL-4RA	4.4 (3.9-4.8)	6.1 (5.1-7.1)	1.367	4.8 x 10^{-4}
SORT1	239 (208-270)	150 (117-184)	-1.688	7.7 x 10^{-4}
PLC	142 (116-167)	178 (154-203)	1.277	9.7 x 10^{-4}
TNF-R1	43.4 (33.6-53.1)	49.9 (41.1-58.9)	1.172	2.7 x 10^{-3}
PTX3	6.2 (5.4-6.9)	7.8 (6.5-9.1)	1.234	2.8 x 10^{-3}
VEGFD	159 (135-184)	129 (107-151)	-1.242	2.9 x 10^{-3}
IL-27	12.1 (9.5-14.7)	9.6 (7.9-11.2)	-1.250	3.2 x 10^{-3}
GLO1	36.2 (25.0-47.5)	61.8 (43.4-80.3)	1.664	4.2 x 10^{-3}
CCL24	64.1 (42.7-85.5)	48.9 (34.2-63.3)	-1.279	4.8 x 10^{-3}
KIM1	345 (217-474)	236 (151-321)	-1.342	5.2 x 10^{-3}

Data are expressed as mean linear ddCq and 95%CI. LLD$^+$/AF$^-$- FH patients under lipid-lowering drugs therapy-only; LLD$^+$/AF$^+$ - patients under combined long-term LDL apheresis/LLD; *Pcorr* value corrected for multiple comparisons (Benjamini–Hochberg correction); FC (fold-change) between group medians of linear ddCq

Table S2 Comparison of LDLR protein concentration between *LDLR* heterozygotes vs homozygotes.

**LDLR protein**	**Mean Linear ddCq (95%CI)**		
**a/ LLD^{+}/AF^{+}**	**Heterozygotes**	**Homozygotes**	***P***
**Before treatment**	31.2 (16.5-45.8)	16.9 (13.9-19.9)	0.01
**After treatment**	14.9 (9.3-20.5)	7.7 (4.4-10.9)	0.05

Data are expressed as mean linear ddCq and 95%CI.

Table S3 Plasma lipid level changes with respect to treatment.

	LLD^{+}/AF^{-}		p value	LLD^{+}/AF^{+}		*P* value
	**before**	**after**		**before**	**after**	
Total Cholesterol	7.5±1.7	4.7±0.7	<0.001	5.3±1.6	1.8±0.4	<0.001
LDL Cholesterol	5.6±1.6	2.8±0.6	<0.001	3.3±1.4	0.6±0.3	<0.001
HDL Cholesterol	1.5±0.4	1.6±0.4	ns	1.3±0.4	0.9±0.2	<0.001
Triglycerides	1.6±0.9	1.5±0.8	ns	1.8±1.3	0.9±0.7	0.002

The data are expressed as the mean ± standard deviation (SD). Bonferroni correction was applied on significance levels. LDL - low density lipoprotein; HDL - high density lipoprotein, Lipid concentration is expressed in mmol/L

Table S4 Classification of protein biomarkers according to biological process. Data were drawn from https://www.olink.com/products/) for biological processes and from www.uniprot.org for the molecular mass of proteins.

**a/ LLD$^+$/AF$^-$**		
**Protein**	**Molecular mass (kDa)**	**Biological processes**
TM	60.3	coagulation, platelet activation, wound healing
DKK-1	28.7	
CCL3	10.1	MAPK cascade
ST2	63.4	inflammatory response
CD4	51	cell adhesion, immune response
PDFG subunit B	27.3	coagulation, heart development, MAPK cascade, platelet activation, regulation of blood pressure, response to hypoxia, response to peptide hormone, wound healing
AGRP	14.4	response to peptide hormone
IL18	22.3	angiogenesis, blood vessel morphogenesis, cell adhesion, immune response, inflammatory response,
THPO	37.8	MAPK cascade
LOX1	31	inflammatory response, proteolysis
**b/ LLD$^+$/AF$^+$**		
**Protein**	**Molecular mass (kDa)**	**Biological processes**
LDLR	95.4	catabolic process,
OPN	35	Wound healing, immune response, inflammatory response
PCSK9	74	catabolic process, proteolysis, response to peptide hormone
MMP-3	54	proteolysis
COL1A1	139	catabolic processes, cell adhesion, coagulation, platelet activation, response to peptide hormone, wound healing
GDF-2	47.3	angiogenesis, blood vessel morphogenesis, MAPK cascade
KLK6	26.8	wound healing, proteolysis
CTRC	29.5	proteolysis
IL-4RA	89.7	immune response, inflammatory response,
SORT1	77-92	response to peptide hormone,
PLC	470	Angiogenesis, blood vessel morphogenesis, catabolic process,
TNF-R1	24-50	inflammatory response
PTX3	42	inflammatory response,
VEGFD	40.4	angiogenesis, blood vessel morphogenesis, response to hypoxia,
IL-27	25.4-27.5	cell adhesion,
GLO1	20.8	inflammatory response
CCL24	13.1	angiogenesis, blood vessel morphogenesis, MAPK cascade, chemotaxis,
KIM1	39.3	inflammatory response
